# A single residue in the VP3 capsid protein governs virulence and informs live-attenuated vaccine design for coxsackievirus A6

**DOI:** 10.1128/jvi.00149-26

**Published:** 2026-04-27

**Authors:** Kexin Liu, Zeyu Liu, Xingyu Yan, Zhenlin Yang, Xue Li, Chao Zhang

**Affiliations:** 1 Shanghai Institute of Infectious Disease and Biosecurity, Fudan University, Shanghai, China; 2 Shanghai Key Laboratory of Lung Inflammation and Injury, Department of Pulmonary Medicine, Zhongshan Hospital, Fudan University 12478, Shanghai, China; 3 Shanghai Engineering Research Center for Synthetic Immunology, Shanghai, China; University of Kentucky College of Medicine, Lexington, Kentucky, USA

**Keywords:** hand, foot, and mouth disease, coxsackievirus A6, virulence determinant, KREMEN1 receptor, vaccine

## Abstract

Coxsackievirus A6 (CVA6) is a major pathogen of hand, foot, and mouth disease, yet the viral determinants of its severe pathogenicity remain undefined, hampering intervention strategies. Here, by comparing a highly lethal clinical isolate (CVA6-HeB) with an attenuated strain (CVA6-TW141), we identified the capsid-encoding P1 region as the primary virulence determinant in neonatal mice. Fine-mapping identified a single critical residue at position 238 of VP3 (VP3-238): glutamic acid (E) in virulent CVA6-HeB versus alanine (A) in attenuated CVA6-TW141. Introducing E238 into the attenuated CVA6-TW141 was sufficient to confer lethality, and conversely, reverting it to alanine in the lethal CVA6-HeB completely abolished virulence, demonstrating that VP3-E238 is necessary and sufficient for lethal disease. E238 is dominant (>93%) in circulating CVA6 strains, suggesting a significant fitness advantage. This residue is situated at the edge of the canyon, a key receptor-binding site. Replacing E238 with alanine (E238A) did not affect virion assembly or growth in cultured cells. However, this mutation drastically reduced binding to the essential murine entry receptor KREMEN1 and attenuated virulence by over 10,000-fold in mice, correlating with significantly lower tissue viral loads and pathology. Based on this mechanism, we rationally designed a live-attenuated vaccine candidate by introducing the attenuating E238A mutation. Immunization with this candidate provided complete protection against a subsequent lethal challenge with a heterologous CVA6 strain. Our study defines VP3-E238 as a key molecular switch for CVA6 pathogenicity and establishes a foundation for rational vaccine development.

**IMPORTANCE:**

Hand, foot, and mouth disease is a common childhood illness increasingly caused by coxsackievirus A6 (CVA6), which can sometimes lead to severe complications. Currently, there are no specific vaccines or treatments available against CVA6. We identified the precise reason for the differing virulence between CVA6 strains. Comparing a lethal strain with a harmless one revealed a single determinant: residue 238 on the VP3 capsid protein. A glutamic acid (“E”) at this site confers virulence, while alanine (“A”) results in attenuation. By engineering an “E” to “A” mutation, we created a virus that is safe in mice but remains immunogenic. This engineered strain, used as a live vaccine, provided complete protection against lethal CVA6 challenge. Our work pinpoints a key virulence switch and presents a direct strategy for developing a safe CVA6 vaccine.

## INTRODUCTION

Hand, foot, and mouth disease (HFMD) is a significant global public health concern, frequently causing widespread outbreaks (1). While historically associated with enterovirus A71 (EV-A71) and coxsackievirus A16 (CVA16) (1), the epidemiological landscape has shifted markedly. Coxsackievirus A6 (CVA6) has now emerged as a major and increasingly prevalent causative agent worldwide (2–5). Notably, CVA6 infections often present with severe and atypical manifestations, such as extensive skin eruptions, onychomadesis, and complications, including meningitis and pulmonary edema, highlighting its considerable pathogenic potential (2, 6). Currently, there are no licensed vaccines or specific therapeutics against CVA6, although several experimental vaccine candidates are under investigation (7–10). This critical gap underscores the urgent need to elucidate its pathogenic mechanisms to enable the development of effective countermeasures.

CVA6 is a positive-sense single-stranded RNA virus with a genome of approximately 7.4 kb (11). Its genome contains a single open reading frame (ORF) that is translated into a large polyprotein, which is subsequently processed into three precursor polypeptides (P1, P2, and P3). The P1 polyprotein is cleaved to form the four structural capsid proteins (VP1, VP2, VP3, and VP4), which assemble into the icosahedral viral particle (10–13). VP1–VP3 are exposed on the viral surface and are critical determinants of antigenicity, receptor engagement, and host tropism. In contrast, the P2 and P3 precursors are processed into non-structural proteins, which facilitate viral replication and modulate host immune responses. CVA6 employs a two-receptor entry mechanism: heparan sulfate proteoglycans (HSPGs) mediate initial attachment, while the major receptor, Kringle-containing transmembrane protein 1 (KREMEN1/KRM1), binds within the canyon region of the capsid to induce uncoating (12, 14). Although the fundamental virological features of CVA6 are established, the specific viral determinants that govern severe *in vivo* pathogenicity remain largely unidentified.

In this study, we systematically investigated the genetic basis of CVA6 virulence by comparing a highly virulent clinical isolate (CVA6-HeB) with an attenuated strain (CVA6-TW141). We hypothesized that specific genetic elements were responsible for their stark difference in lethality in neonatal mice. To test this, we constructed chimeric viruses, performed site-directed mutagenesis, and conducted *in vivo* pathogenesis screening. This approach allowed us to map the primary virulence determinant to the P1 capsid region and to identify a single critical residue (VP3-238) that dictates lethality. We further characterized its impact on receptor binding and *in vivo* replication. Ultimately, based on this finding, we designed and validated a rationally engineered live-attenuated vaccine candidate. Our work elucidates a key molecular mechanism of CVA6 pathogenicity and supports the rational design of vaccines against this virus.

## RESULTS

### The CVA6 strains TW141 and HeB exhibit similar replication kinetics *in vitro* but profoundly different lethality *in vivo*

**Fig 1:**
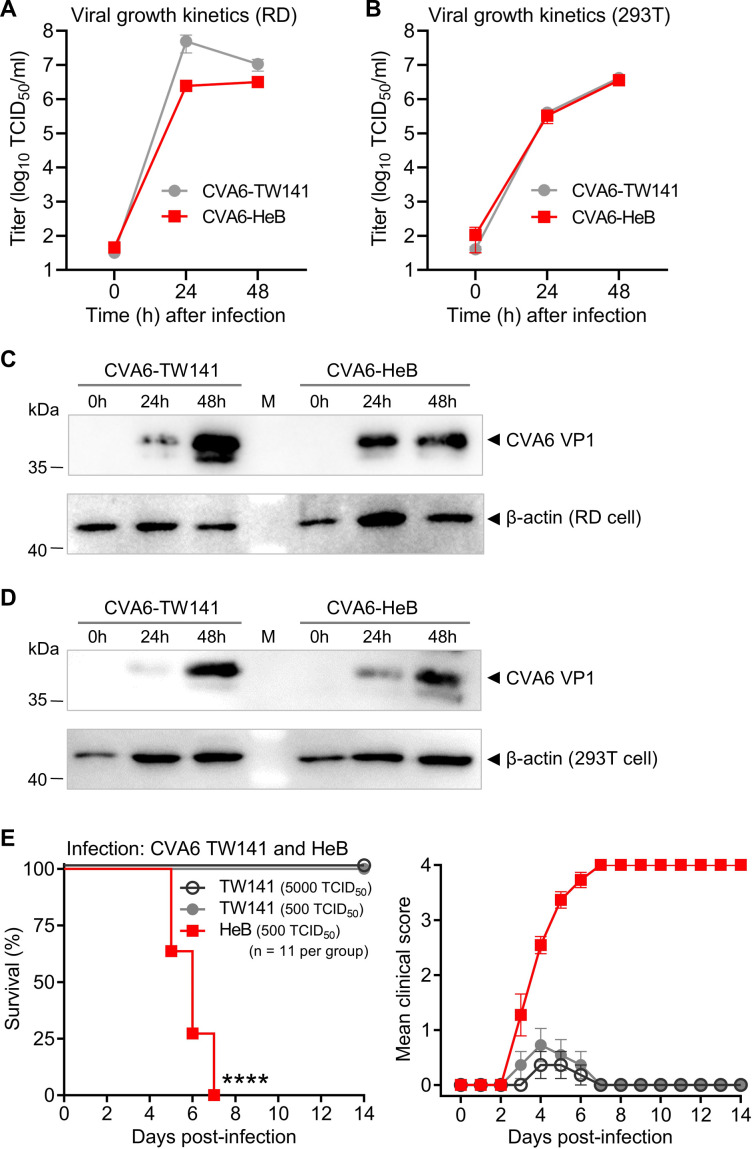
CVA6-HeB, but not CVA6-TW141, is lethal in neonatal mice despite their similar replication kinetics *in vitro*. (**A**) Replication kinetics in RD cells. RD cells were infected with CVA6-TW141 or CVA6-HeB at an MOI of 0.1. Viral titers were determined by TCID_50_ assay at 0, 24, and 48 hpi. Data represent mean ± SEM from three biological replicates. In this study, CVA6-TW141 was rescued from an infectious clone, while CVA6-HeB is a clinical isolate. (**B**) Replication kinetics in 293T cells. 293T cells were infected and analyzed as in (**A**). (**C**) CVA6 VP1 protein expression in RD cells. RD cells were infected with each virus at an MOI of 0.1. Cell lysates were immunoblotted with anti-VP1 antibody. Anti-β-actin antibody was used as a loading control. M, protein marker. (**D**) CVA6 VP1 protein expression in 293T cells. 293T cells were infected and analyzed as in (**C**). (**E**) Survival analysis in a neonatal mouse model. Two-day-old ICR mice (*n* = 11 per group) were inoculated intraperitoneally (i.p.) with either CVA6-TW141 (5,000 or 500 TCID_50_/mouse) or CVA6-HeB (500 TCID_50_/mouse). Survival and clinical signs were monitored for 14 days. Clinical scores were assigned as: 0, healthy; 1, reduced mobility; 2, limb weakness; 3, limb paralysis; 4, death. Statistical significance was determined by the Log-rank (Mantel–Cox) test (****, *P* < 0.0001). Error bars indicate SEM.

Our study utilized two CVA6 strains: CVA6-TW141 (from Taiwan), a strain rescued from an infectious clone (15), and CVA6-HeB (from Hebei Province), a clinical isolate (16). We first compared their replication efficiency in permissive cell lines. RD and 293T cells were infected with each virus at a multiplicity of infection (MOI) of 0.1. Both strains replicated with highly similar kinetics, yielding comparable viral titers at 24 and 48 h post-infection (hpi) (Fig. 1A and B). Consistent with this, western blot analysis revealed similar levels of the major capsid protein VP1 in cells infected with either strain at these time points (Fig. 1C and D).

We next compared the pathogenicity of both strains in a neonatal mouse model. Two-day-old ICR mice were inoculated i.p. with CVA6-TW141 (500 or 5,000 TCID_50_/mouse) or CVA6-HeB (500 TCID_50_/mouse) and monitored for survival and clinical signs for 14 days. Despite their similar replication in cultured cells, the two strains showed dramatically different outcomes *in vivo*. Infection with CVA6-HeB induced progressive limb weakness and paralysis, leading to 100% mortality. In contrast, CVA6-TW141 caused no mortality or severe clinical signs, even at the 10-fold higher challenge dose (5,000 TCID_50_/mouse) (Fig. 1E).

### The P1 capsid region is the primary determinant of CVA6 virulence in mice

**Fig 2:**
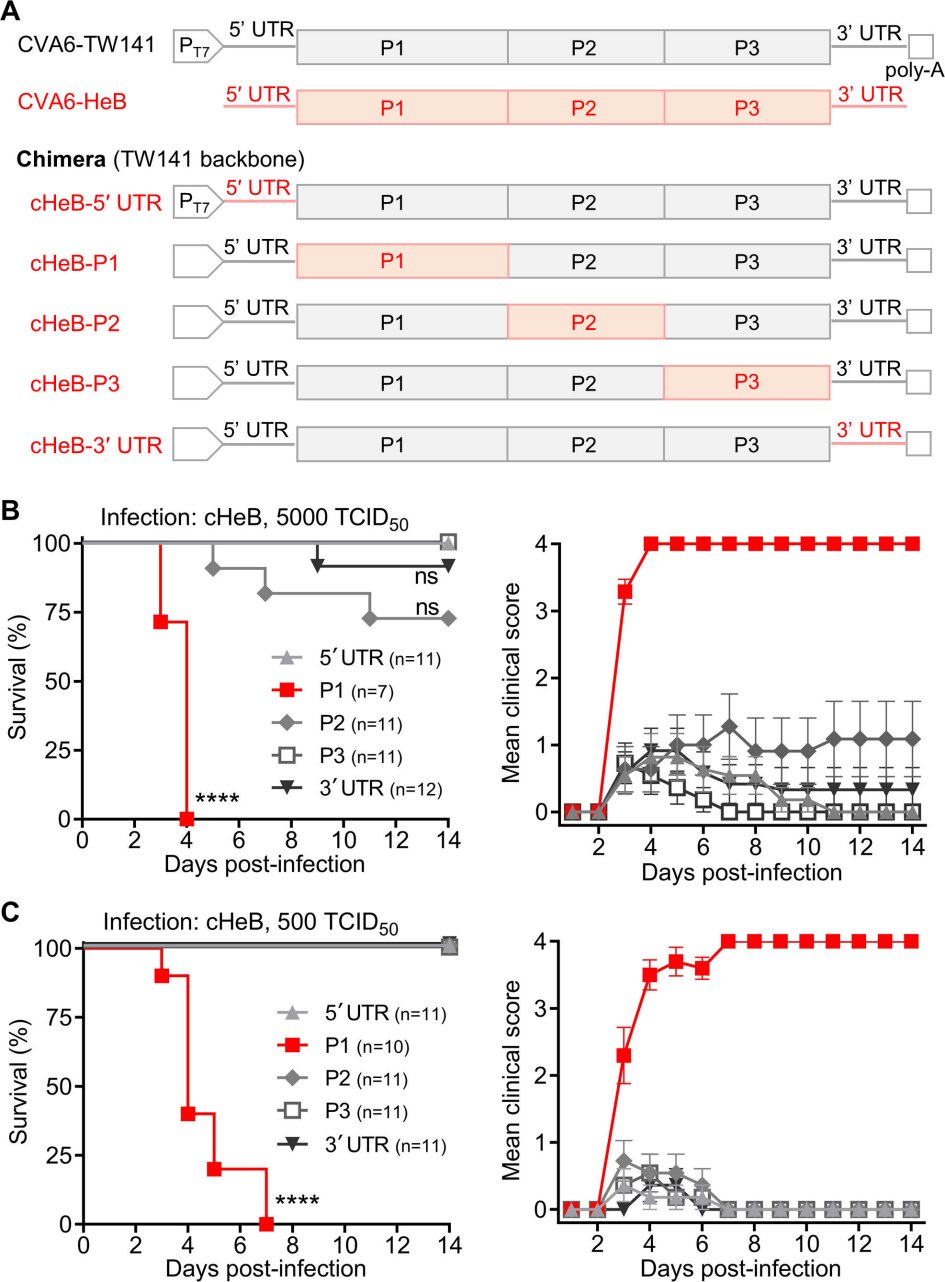
The P1 capsid region is the primary determinant of CVA6 virulence in neonatal mice. (**A**) Schematic representation of chimeric infectious clones generated on the non-lethal CVA6-TW141 backbone. Individual genomic regions (5′ UTR, P1 capsid, P2, P3, or 3′ UTR) were replaced with the corresponding segments from the virulent clinical isolate CVA6-HeB. (B–C) Survival of mice challenged with chimeric CVA6 viruses. Two-day-old ICR mice were inoculated i.p. with (**B**) 5,000 TCID_50_ per mouse or (**C**) 500 TCID_50_ per mouse of the indicated chimeric virus. Survival and clinical signs were monitored for 14 days. Clinical scoring criteria are as described in Fig. 1. Group sizes (*N*) are indicated. Statistical significance was determined by the Log-rank (Mantel–Cox) test, with all groups compared against the non-lethal cHeB-5′ UTR group. ns, not significant, *P* > 0.05; ****, *P* < 0.0001. Error bars represent SEM.

To map the virulence determinant of CVA6-HeB, we generated a panel of chimeric viruses on the non-lethal CVA6-TW141 backbone, replacing individual genomic regions (5′ UTR, P1 capsid, P2, P3, or 3′ UTR) with the corresponding segments from the virulent CVA6-HeB strain. The resulting viruses were designated cHeB-5′UTR, cHeB-P1, cHeB-P2, cHeB-P3, and cHeB-3′UTR, respectively (Fig. 2A). All chimeras were successfully rescued, sequence-verified, amplified, and titrated for *in vivo* studies.

We next assessed the pathogenicity of each chimera in neonatal mice. At a high challenge dose (5,000 TCID_50_/mouse), only cHeB-P1 caused 100% mortality. Chimeras carrying the HeB-derived P2 or 3′ UTR regions induced partial lethality (27.3% and 8.3%, respectively), while cHeB-5′UTR and cHeB-P3 were non-lethal (Fig. 2B). When the challenge dose was reduced to 500 TCID_50_/mouse, cHeB-P1 again resulted in complete mortality, whereas all other chimeras caused no deaths and only mild, transient clinical signs (Fig. 2C). Together, these results demonstrate that the P1 capsid region is both necessary and sufficient for the lethal phenotype of CVA6-HeB, establishing it as the primary genetic determinant of CVA6 virulence.

### A single capsid residue is necessary and sufficient for CVA6 virulence

**Fig 3:**
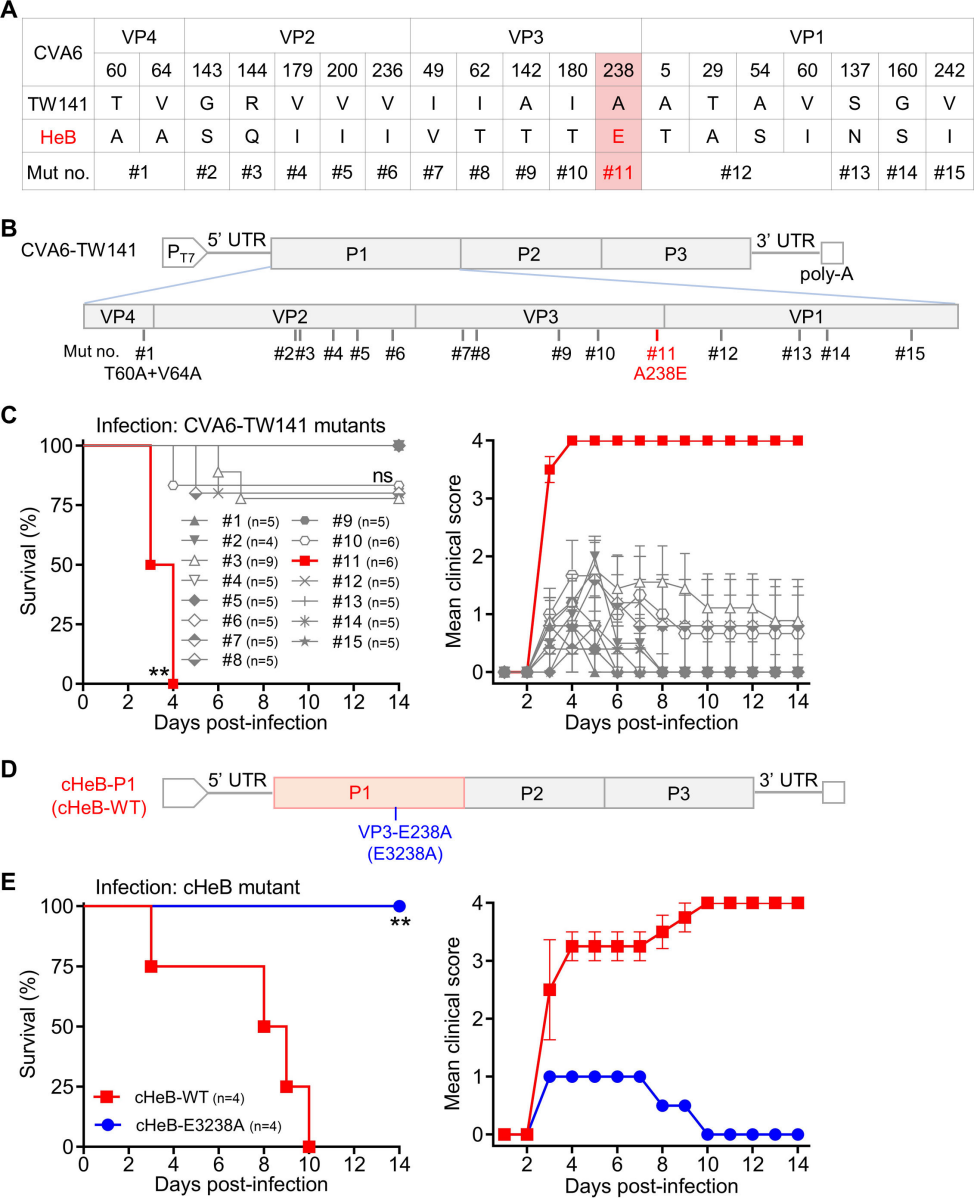
Glutamic acid (**E**) at position 238 of the VP3 capsid protein is a critical virulence determinant of CVA6. (**A**) Summary of amino acid differences and mutagenesis strategy in the P1 region. Table listing the 19 divergent residues between CVA6-TW141 and CVA6-HeB among the capsid proteins (VP4, VP2, VP3, and VP1). To map virulence determinants, 15 mutant viruses (#1 to #15) were generated on the CVA6-TW141 backbone by substituting individual or combined residues with those from CVA6-HeB. Mutant #1 contains a double substitution in VP4. Mutant #12 is a combination mutant of four residues in the VP1 N-terminus. The remaining mutants represent single-amino-acid changes. Mutant #11 (VP3-A238E) is highlighted in red. (**B**) Schematic of the CVA6 infectious clone and mutant construction. Diagram of the T7 promoter-driven CVA6-TW141 infectious clone, illustrating the genomic locations where the amino acid substitutions corresponding to mutants #1 to #15 (detailed in panel A) were introduced. The site of the key mutation #11 (VP3-A238E) is indicated in red. (**C**) *In vivo* virulence screening of point mutants. Two-day-old ICR mice (*n* = 4–9 per group) were inoculated i.p. with 500 TCID_50_ per mouse of each indicated CVA6-TW141 mutant virus (#1 to #15). Survival and clinical signs were monitored for 14 days. Clinical scoring criteria are as described in Fig. 1. Only mutant #11 (VP3-A238E) induced 100% mortality, whereas the other mutants caused no or only partial lethality. Statistical significance versus the non-lethal mutant #1 group was determined by the Log-rank (Mantel–Cox) test. ns, *P* > 0.05; **, *P* < 0.01. Error bars represent SEM. (**D**) Introduction of the VP3-E238A (E3238A) mutation into the virulent chimera cHeB-P1. Schematic of the site-directed mutagenesis performed to generate the E3238A substitution within the fully virulent chimeric clone cHeB-P1 (hereafter referred to as cHeB-WT), which was constructed and characterized in Fig. 2. (**E**) The E3238A substitution completely abolished the lethality of cHeB-WT. Two-day-old ICR mice (*n* = 4 per group) were inoculated i.p. with 500 TCID_50_ per mouse of the virulent chimera cHeB-WT and the point mutant cHeB-E3238A. Survival was monitored for 14 days. Statistical significance was determined by the Log-rank (Mantel–Cox) test. **, *P* < 0.01. Error bars represent SEM.

To identify the specific residue(s) responsible for the lethal phenotype, we compared the capsid sequences of CVA6-HeB and CVA6-TW141, revealing 19 divergent amino acids (Fig. 3A). To map virulence determinants, we designed a panel of 15 mutants on the CVA6-TW141 backbone (Fig. 3A and B). Given their internal location within the virion, the divergent residues in VP4 (mutant #1) and the VP1 N-terminus (mutant #12) were substituted as clusters. The remaining divergent sites were individually mutated, each generating a single-point mutant (Fig. 3A and B). All 15 mutants (#1 to #15) were rescued, sequence-verified, amplified, and titrated prior to systematic *in vivo* screening.

Neonatal mice were inoculated with 500 TCID_50_ of each mutant and monitored for survival. Strikingly, only mutant #11, which carries a single alanine-to-glutamic acid substitution at residue 238 of VP3 (A238E), caused 100% mortality. All other mutants showed either low-level lethality (<25%) or no lethality (Fig. 3C).

To test whether VP3-E238 is required for CVA6 virulence, we used the virulent cHeB-P1 chimera (designated cHeB-WT), which was previously shown in Fig. 2 to be fully lethal. We introduced a VP3-E238A substitution (numbered E3238A according to the enterovirus structural biology convention, where VP3 residues start at 3001) into this backbone (Fig. 3D). The resulting virus, cHeB-E3238A, was confirmed by sequencing of the entire capsid region to contain no unintended mutations outside the introduced substitution. This cHeB-E3238A virus was completely avirulent: all infected mice survived with no severe clinical signs, in stark contrast to the 100% mortality caused by cHeB-WT (Fig. 3E).

Together, these results establish that a single glutamic acid at VP3-238 is both necessary and sufficient to confer the lethal phenotype of CVA6 in neonatal mice.

### Glutamic acid (E) at VP3-238 is the dominant natural variant and confers full virulence to CVA6

**Fig 4:**
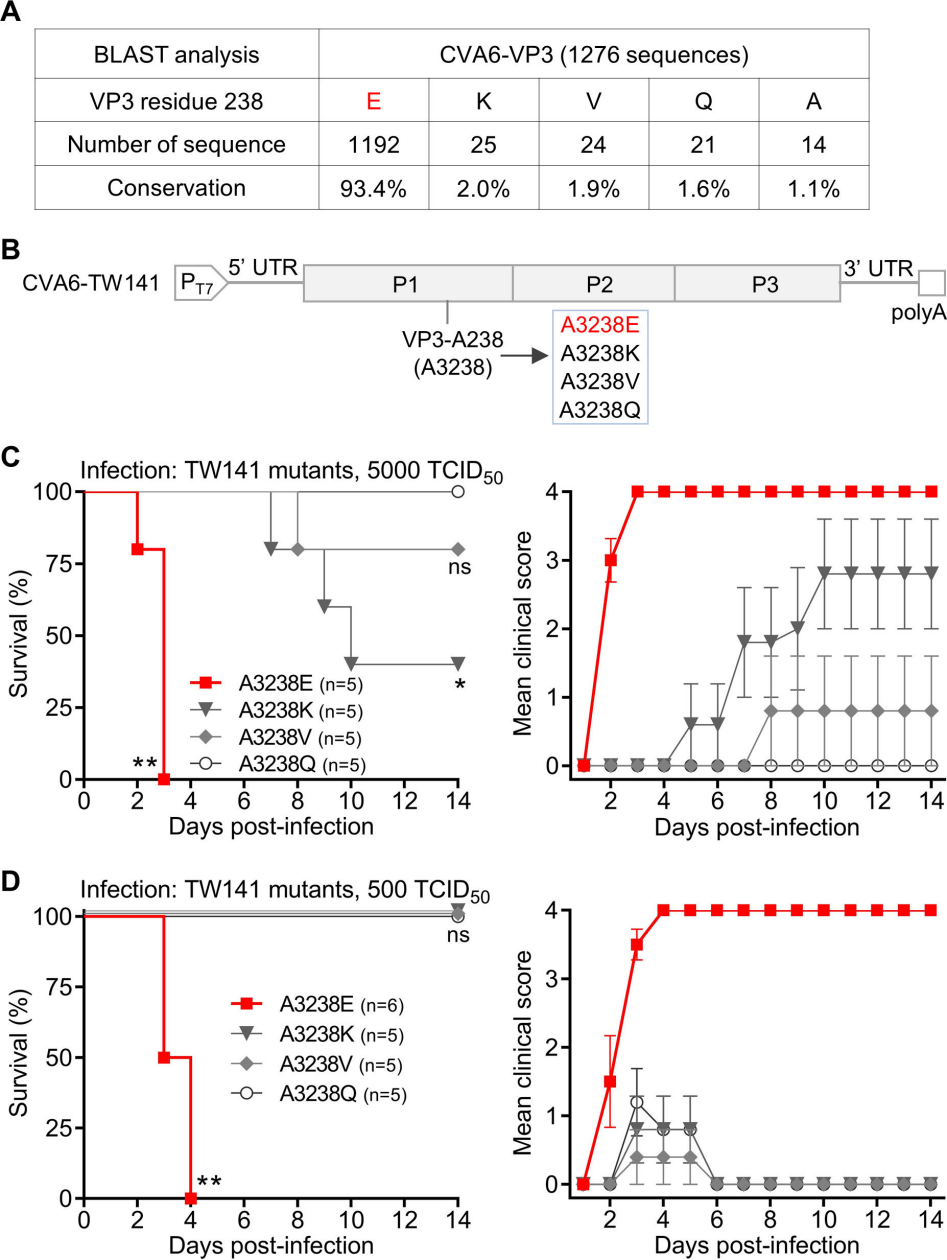
Glutamic acid (E) at VP3-238 is the dominant natural variant and confers full virulence to CVA6. (**A**) Natural variation at residue 238 in CVA6 VP3. Analysis of 1,276 CVA6 VP3 sequences from GenBank (as of August 2025) revealed glutamic acid (E) as the predominant residue at position 238, highlighted in red. Other naturally occurring variants include lysine (K), valine (V), glutamine (Q), and alanine (**A**). (**B**) Construction of variant viruses at VP3-238. Schematic of the T7 promoter-driven CVA6-TW141 infectious clone, showing the introduction of four point-mutations to reproduce the natural variants at VP3-238: A3238E, A3238K, A3238V, and A3238Q. The A3238E mutant, highlighted in red, corresponds to the previously identified virulent mutant #11 (Fig. 3A through C). (**C and D**) Virulence assessment of VP3-238 variants. Two-day-old ICR mice (*n* = 5–6 per group) were inoculated i.p. with (**C**) 5,000 or (**D**) 500 TCID_50_ per mouse of the indicated mutant viruses. Survival and clinical signs were monitored for 14 days (scoring as in Fig. 1). Statistical significance was determined by the Log-rank (Mantel–Cox) test, with each group compared against the non-lethal A3238Q mutant. ns, *P* > 0.05; *, *P* < 0.05; **, *P* < 0.01. Error bars represent SEM.

Having identified VP3-E238 as a critical virulence determinant, we sought to understand the relevance of this residue in naturally circulating CVA6 strains. Analysis of 1,276 CVA6 VP3 sequences identified five naturally occurring amino acids at position 238, with glutamic acid (E) being overwhelmingly predominant (93.4%); lysine (K), valine (V), glutamine (Q), and alanine (A) were also present (Fig. 4A). To determine whether other natural variants could support virulence, we used site-directed mutagenesis to replace the native alanine (A) at VP3-238 in the non-lethal CVA6-TW141 backbone with each of the other natural variants, generating the corresponding mutant viruses A3238E, A3238K, A3238V, and A3238Q (Fig. 4B).

Neonatal mice were inoculated with high (5,000 TCID_50_/mouse) or low (500 TCID_50_/mouse) doses of each mutant. At the high dose, only the A3238E mutant, which is the VP3-A238E variant previously identified as the lethal mutant #11 in Fig. 3C, caused complete (100%) mortality. The A3238K and A3238V mutants induced partial lethality, while A3238Q was non-lethal (Fig. 4C). At the more stringent low dose (500 TCID_50_/mouse), A3238E remained fully lethal, whereas all other variants (A3238V, A3238K, and A3238Q) caused no mortality, with infected animals exhibiting only mild or transient clinical signs (Fig. 4D).

These results demonstrate that among all naturally occurring residues at VP3-238, only glutamic acid (E) supports the complete lethal phenotype, which is maintained even at a low challenge dose.

### The E3238A mutation does not impair basic virological properties of CVA6 *in vitro*

**Fig 5:**
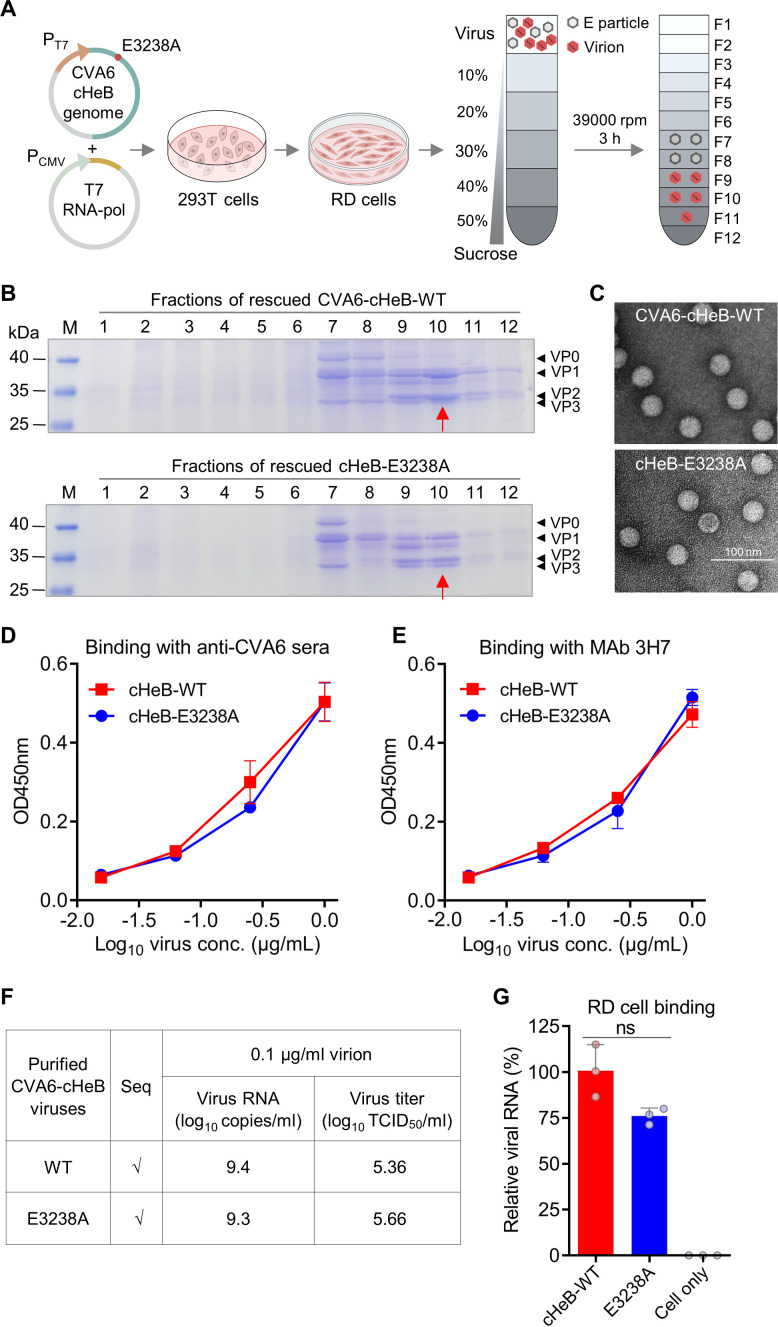
The E3238A mutation does not alter the basic *in vitro* virological properties of CVA6. (**A**) Schematic of virus production and purification. The cHeB-E3238A mutant (previously constructed and validated in Fig. 3D and E) and its parental cHeB-WT virus were rescued in 293T cells, amplified in RD cell cultures (500 mL each), and purified by sucrose gradient ultracentrifugation. E particle, empty particle. (**B**) Protein composition of purified CVA6 virions. Sucrose gradient fractions from CVA6 cHeB-WT or cHeB-E3238A preparations were analyzed by SDS-PAGE and stained with Coomassie blue, showing the profile of structural proteins. Fraction #10 (red arrow), enriched for mature virions, was used for subsequent experiments. (**C**) Morphology of purified CVA6 virions. Representative negative-stain electron microscopy images of purified CVA6 cHeB-WT and cHeB-E3238A virions from fraction #10. Scale bar, 100 nm. (D–E) Antigenic profile of purified CVA6 virions. ELISA assessing reactivity of serially diluted CVA6 cHeB-WT and cHeB-E3238A virions with (**D**) polyclonal anti-CVA6 mouse serum or (**E**) the neutralizing MAb 3H7. Data are mean ± SEM of triplicate wells. Conc., concentration. (**F**) Comparable viral RNA copy number and infectious titer. Purified CVA6 cHeB-WT and cHeB-E3238A virions were diluted to an equal protein concentration (0.1 µg/mL). Viral genome copy number was determined by absolute RT-qPCR using a plasmid standard. Infectious titer was measured by the TCID_50_ assay. (**G**) Comparable cell binding capacity. RD cells were incubated with equal genome copies (1 × 10⁹/mL) of CVA6 cHeB-WT or cHeB-E3238A virions at 4°C. Cell-bound viral RNA was quantified by RT-qPCR and normalized to cellular β-actin mRNA. Data are mean ± SD of three biological replicates. The Mann–Whitney test was used to compare differences. ns, not significant, *P* > 0.05.

Given the fully attenuated phenotype of the cHeB-E3238A mutant in neonatal mice (Fig. 3E), we investigated whether this attenuation resulted from defects in fundamental viral characteristics. We first purified cHeB-WT and cHeB-E3238A virions from infected RD cell lysates and supernatants by sucrose gradient ultracentrifugation (Fig. 5A). SDS-PAGE analysis of gradient fractions showed that both viruses exhibited identical protein composition, with structural proteins co-sedimenting in the same fractions (primarily #7 to #10) (Fig. 5B). The fraction enriched for mature virions (fraction #10) was used for subsequent analyses. Negative-stain electron microscopy confirmed that purified preparations from both cHeB-WT and E3238A viruses contained predominantly intact, spherical particles approximately 30 nm in diameter, characteristic of enteroviruses (Fig. 5C).

Antigenic analysis by ELISA revealed comparable, dose-dependent binding of both cHeB-WT and cHeB-E3238A to anti-CVA6 polyclonal serum and to the conformation-sensitive neutralizing monoclonal antibody (MAb) 3H7 (12) (Fig. 5D and E). These results indicate that the E3238A mutation does not alter the overall antigenic structure.

To compare infectivity, purified cHeB-WT and cHeB-E3238A virions were diluted to an equal protein concentration (0.1 µg/mL). Viral genome copy number, quantified by absolute quantitative reverse transcription PCR (RT-qPCR), and infectious titer, measured by TCID_50_ assay, were similar between the two viruses (Fig. 5F), demonstrating an unaltered physical-to-infectious particle ratio.

Finally, to assess cell attachment, RD cells were incubated with equal genome copies (1  ×  10⁹ copies/mL) of each virus at 4°C. Subsequent RT-qPCR analysis of cell-associated viral RNA revealed no significant difference in attachment between cHeB-WT and cHeB-E3238A (Fig. 5G).

Collectively, these data demonstrate that the E3238A mutation does not significantly alter virion assembly, morphology, antigenicity, infectivity, or cell-binding capability. The profound attenuation of this mutant *in vivo* is therefore not attributable to major defects in these core virological properties.

### The E3238A mutation abolishes CVA6-induced mortality by reducing viral loads and attenuating tissue pathology

**Fig 6:**
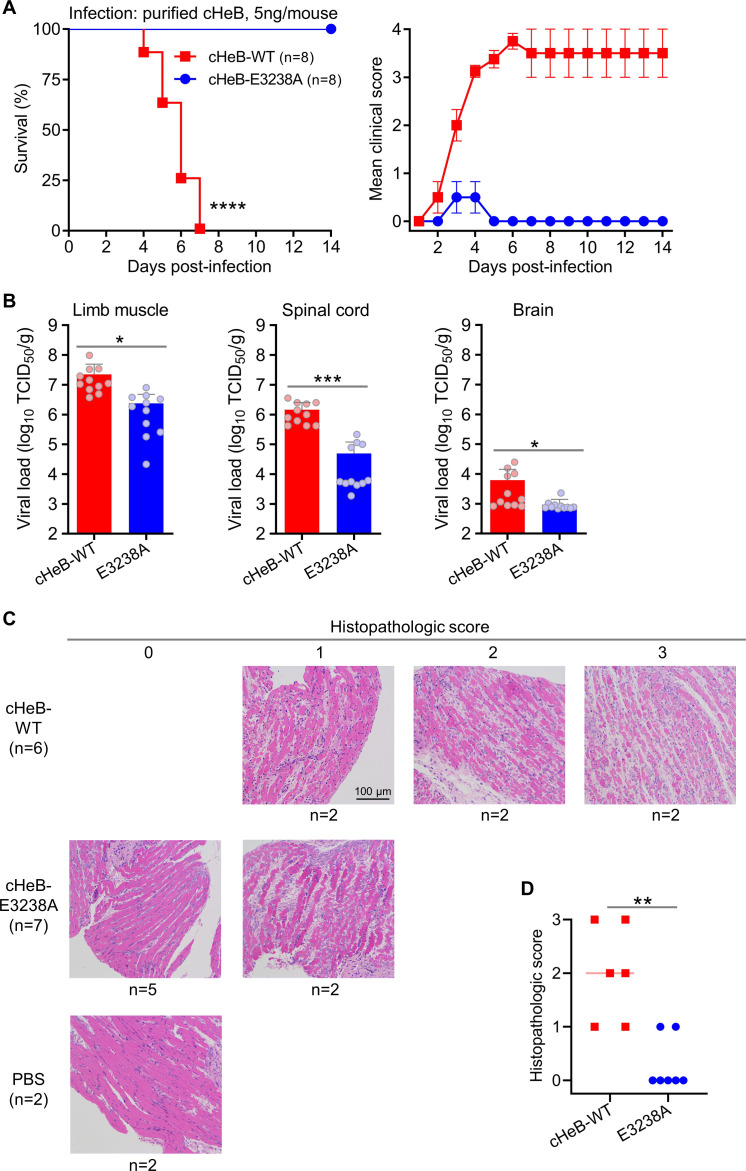
The E3238A mutation abolishes CVA6-induced mortality by reducing viral loads and attenuating tissue pathology in neonatal mice. (**A**) The E3238A mutation abolishes virus-induced mortality and clinical disease. Two-day-old ICR mice (*n* = 8 per group) were inoculated i.p. with 5 ng per mouse of purified CVA6 cHeB-WT or cHeB-E3238A virions. Survival (left) and clinical scores (right) were monitored for 14 days. Statistical significance was determined by the Log-rank (Mantel–Cox) test. ****, *P* < 0.0001. Error bars represent SEM. (**B**) The E3238A mutation significantly lowers viral loads in the limb muscle, spinal cord, and brain. Viral titers were quantified by TCID_50_ assay in tissue homogenates at 4 days post-infection (dpi; *n* = 11 per group) and expressed as TCID_50_ per gram of tissue (TCID_50_/g). Each symbol represents one mouse. Data were analyzed by a two-tailed unpaired t-test. *, *P* < 0.05; ***, *P* < 0.001. (**C**) Limb muscle pathology is attenuated by the E3238A mutation. Representative H&E-stained sections of hind limb muscle from mice inoculated with cHeB-WT, cHeB-E3238A, or PBS (control) at 4 dpi. Pathology scores: 0, normal; 1, mild; 2, moderate; 3, severe myositis. Scale bar, 100 μm. Sample sizes (*N*) are provided. Data in panels B and C are from two independent experiments. (**D**) Quantification confirms reduced muscle pathology in E3238A-infected mice. Histopathological scores for individual mice from (**C**). Each symbol represents one mouse. Statistical significance was determined by the Mann–Whitney test. **, *P* < 0.01.

To determine the pathogenic impact of the E3238A mutation, neonatal mice were inoculated with an equal dose (5 ng per mouse) of purified cHeB-WT and cHeB-E3238A virions. All mice infected with cHeB-WT developed progressive limb weakness and paralysis, resulting in 100% mortality. In contrast, all cHeB-E3238A-infected mice survived, displaying only mild and transient clinical signs (Fig. 6A).

We next measured viral loads in target tissues at 4 dpi. The cHeB-E3238A mutant showed significantly lower titers than cHeB-WT in all tissues examined (Fig. 6B). Viral titers in limb muscle were reduced by approximately 14-fold (geometric mean titer: cHeB-WT, 1.41  ×  10⁷ TCID_50_/g; E3238A, 1.03  ×  10⁶ TCID_50_/g). The reduction was most striking in the spinal cord, with a 74-fold decrease (cHeB-WT, 1.10  ×  10⁶ TCID_50_/g; E3238A, 1.48  ×  10⁴ TCID_50_/g). Brain viral loads were also significantly lower, showing about a threefold reduction (cHeB-WT, 2.75  ×  10³ TCID_50_/g; E3238A, 8.69  ×  10² TCID_50_/g) (Fig. 6B).

Consistent with the virological data, histopathological analysis of limb muscle at 4 dpi revealed extensive myofiber necrosis in cHeB-WT-infected mice, whereas muscle architecture in the cHeB-E3238A group remained largely intact (Fig. 6C). Quantitative scoring confirmed that muscle pathology was significantly attenuated in E3238A mutant-infected animals (Fig. 6D).

These results demonstrate that the E3238A mutation markedly impairs viral replication in target tissues and reduces associated pathology, thereby providing a mechanistic explanation for its complete attenuation of lethality *in vivo*.

### The E3238A mutation attenuates CVA6 virulence by over 10,000-fold and impairs binding to the murine receptor KRM1

**Fig 7:**
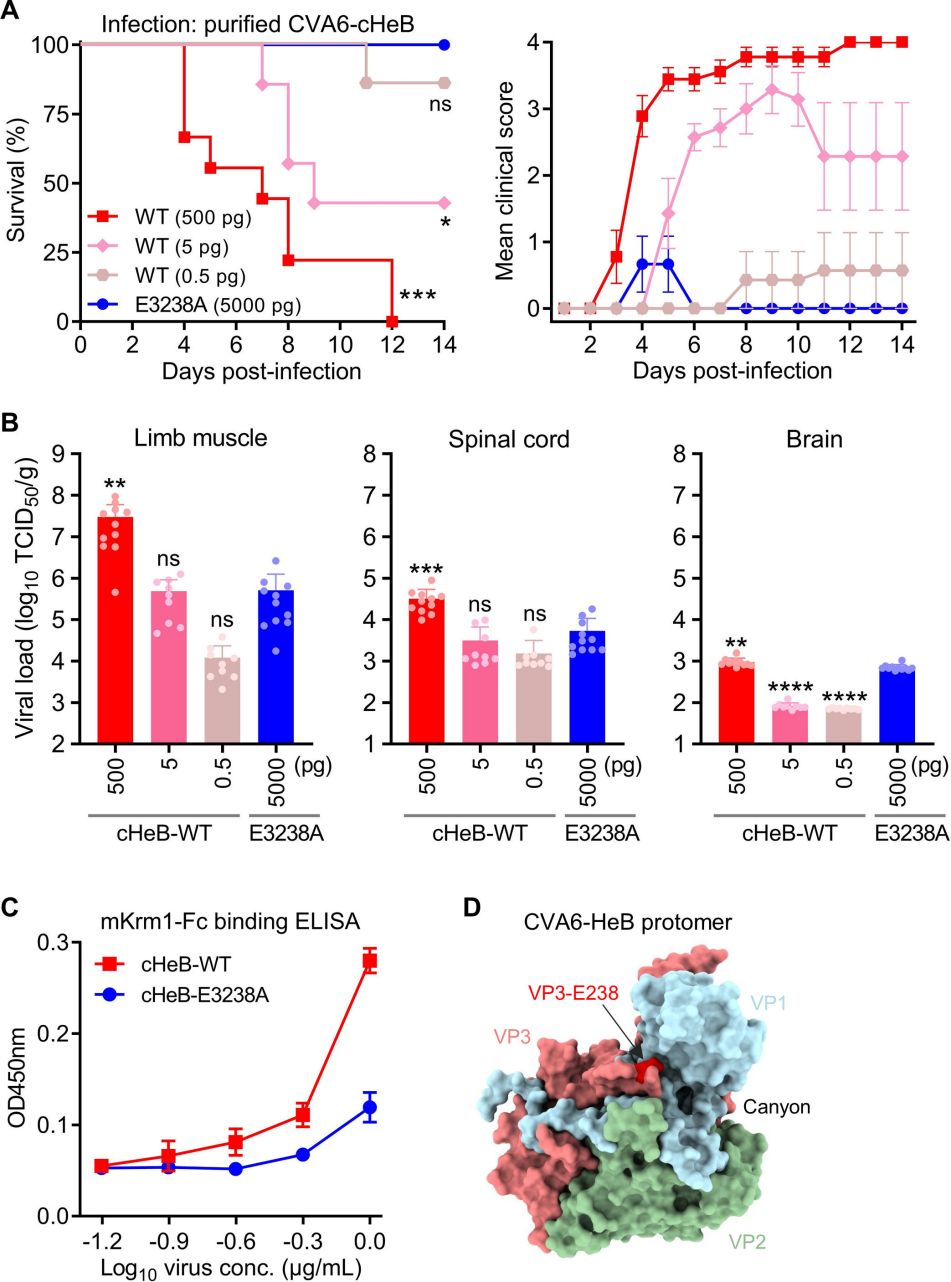
The E3238A mutation attenuates CVA6 virulence by over 10,000-fold and impairs binding to murine KRM1 receptor. (**A**) Survival and clinical disease following CVA6 infection. Two-day-old ICR mice (*n* = 6–9 per group) were inoculated i.p. with serial dilutions of purified cHeB-WT virions or with a single high dose of the cHeB-E3238A mutant. Survival (left) and clinical scores (right) were monitored for 14 days. The cHeB-WT virus exhibited a clear dose-dependent increase in lethality and disease severity, while the cHeB-E3238A mutant showed no lethality or severe clinical signs. Statistical significance was determined by the Log-rank (Mantel–Cox) test, with each group compared against the E3238A mutant. ns, *P* > 0.05; *, *P* < 0.05; ***, *P* < 0.001. Error bars represent SEM. (**B**) Analysis of tissue viral loads at 4 dpi. Viral titers were quantified by TCID_50_ assay in homogenates of limb muscle, spinal cord, and brain from infected mice (*n* = 9–11 per group) and expressed as TCID_50_ per gram of tissue (TCID_50_/g). Each symbol represents an individual mouse. Data were analyzed by a two-tailed unpaired t-test, comparing each cHeB-WT dose group to the cHeB-E3238A group. ns, *P* > 0.05; **, *P* < 0.01; ***, *P* < 0.001; ****, *P* < 0.0001. (**C**) Binding of purified CVA6 virions to the murine receptor KRM1 (mKrm1) was analyzed by ELISA. Serially diluted purified cHeB-WT and cHeB-E3238A virions were coated onto ELISA plates and incubated with mKrm1-Fc. Binding was detected with an HRP-conjugated anti-human Fc antibody. Data represent means ± SD of three replicate samples. (**D**) Structural localization of VP3-E238 in the CVA6 capsid. Surface representation of the CVA6-HeB protomer (PDB: 9VFQ), with VP1 in light blue, VP2 in dark sea green, and VP3 in light coral. VP3-E238 is highlighted in red.

To quantify the extent of attenuation conferred by the E3238A mutation, we performed a dose–response study. Neonatal mice were inoculated with serial dilutions of the virulent cHeB-WT virus (500, 5, or 0.5 pg/mouse) or with a single high dose of the cHeB-E3238A mutant (5,000 pg/mouse) and monitored for 14 days. cHeB-WT exhibited clear dose-dependent lethality, with mortality rates of 100%, 57.1%, and 14.3% at 500, 5, and 0.5 pg, respectively (Fig. 7A). In stark contrast, all mice receiving the high-dose cHeB-E3238A (5,000 pg) survived (Fig. 7A). The E3238A mutation attenuates CVA6 virulence by over 10,000-fold, as the mutant is completely avirulent even at 5,000 pg, a dose 10,000 times higher than the 0.5 pg dose at which the wild-type virus still causes lethality.

We next quantified viral loads in target tissues at 4 dpi (Fig. 7B). Viral titers of cHeB-WT in both limb muscle and spinal cord decreased in a clear dose-dependent manner. Strikingly, the viral loads achieved by the high-dose cHeB-E3238A mutant (5,000 pg) in these tissues were significantly lower than those of the lethal high-dose cHeB-WT (500 pg), and were comparable to the levels seen with the sub-lethal cHeB-WT doses (5 pg and 0.5 pg). This demonstrates a severely restricted replication capacity of the E3238A mutant *in vivo*. In the brain, viral replication was minimal overall, with low titers detected among all groups.

Given the critical role of receptor interaction in infectivity, we tested whether the E3238A mutation affected binding to the murine entry receptor KRM1 (mKrm1). ELISA demonstrated that purified cHeB-WT virions bound mKrm1-Fc with markedly stronger signals than the cHeB-E3238A mutant (Fig. 7C). This indicates that the VP3-A238 residue confers weaker binding activity to mKrm1 compared to E238, which partially explains the significantly reduced replication capacity of the E3238A mutant in neonatal mouse tissues (Fig. 6B and 7B).

To localize VP3-E238, we performed structural mapping using the capsid protomer of CVA6-HeB mature virion (PDB: 9VFQ) (12), which revealed its position within the VP3 C-terminus (Fig. 7D). This residue resides at the edge of the canyon region, a surface depression accessible for engagement with the host receptor KRM1 (12).

Together, these results demonstrate that the E3238A mutation attenuates CVA6 virulence by >10,000-fold. This attenuation is linked to lower viral loads in tissues and to weakened binding to the murine KRM1 receptor.

### The CVA6-cHeB-E3238A mutant elicits neutralizing antibodies and confers complete protection as a live-attenuated vaccine candidate in neonatal mice

**Fig 8:**
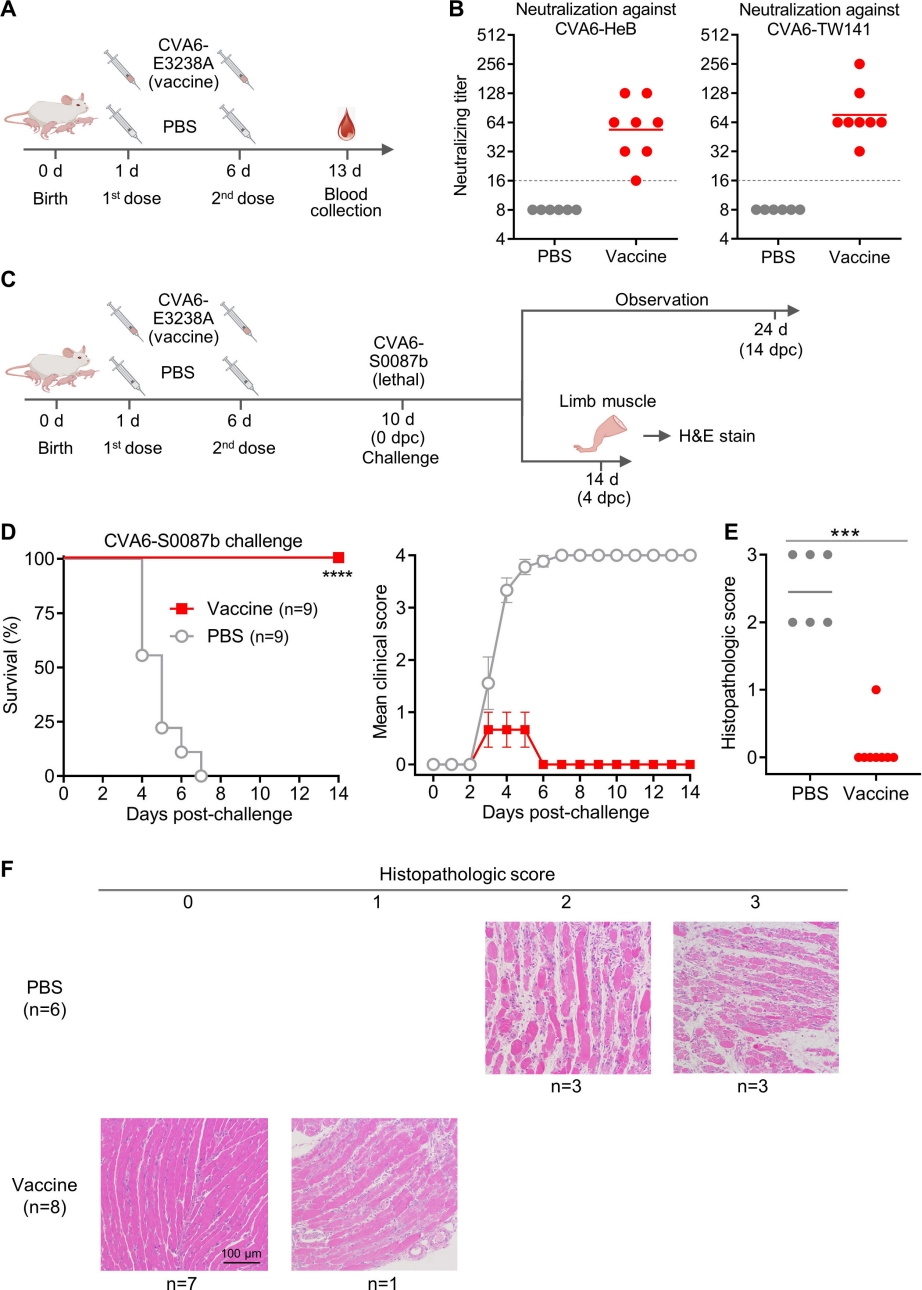
The CVA6 cHeB-E3238A attenuated vaccine elicits neutralizing antibodies and confers complete protection against lethal CVA6 challenge in mice. (**A**) Schematic of the immunization schedule for neutralizing antibody assessment. One-day-old ICR mice were primed i.p. with purified cHeB-E3238A vaccine (0.5 ng/pup) or PBS (control). A booster immunization (5 ng/pup) was administered on day 6. Blood was collected on day 13 for neutralization assays. (**B**) Neutralizing antibody titers against CVA6-HeB and CVA6-TW141. The dashed line indicates the lowest serum dilution tested (1:16). Control sera showed no neutralization at 1:16 and were assigned a titer of 8 for GMT calculation. Each symbol represents an individual mouse, and the solid line indicates the GMT of the group. (**C**) Schematic of the vaccination and challenge experiment. Immunization was performed as described in (**A**). On day 10, mice were challenged with a lethal dose of the virulent CVA6-S0087b strain. Mice for survival analysis (panel D) were monitored for 14 days. For histological analysis (panels E and F), separate groups of mice were sacrificed at 4 days post-challenge (dpc) for tissue collection. (**D**) Vaccination with CVA6-E3238A provides complete protection against lethality and severe disease. Survival (left) and clinical scores (right) were monitored for 14 days after CVA6-S0087b challenge (scoring as in Fig. 1). Statistical significance was determined by the log-rank (Mantel–Cox) test. ****, *P* < 0.0001. Error bars represent SEM. (**E and F**) Vaccination with CVA6-E3238A markedly attenuates challenge-induced muscle pathology. (**E**) Quantification of muscle pathology scores. Each symbol represents one animal. Statistical significance was determined by the Mann–Whitney test. ***, *P* < 0.001. (**F**) Representative H&E-stained limb muscle sections from mice at 4 dpc. Myositis severity was scored as: 0 (absent), 1 (mild), 2 (moderate), and 3 (severe). Scale bar, 100 μm.

Based on the profound attenuation of the cHeB-E3238A mutant (Fig. 6 and 7), we next evaluated its vaccine potential. First, neonatal mice were immunized with cHeB-E3238A on day 1 (0.5 ng) and day 6 (5 ng), while the control group received PBS. Blood samples were collected 1 week after the booster immunization, and neutralizing antibody titers were determined by neutralization assay (Fig. 8A). The results showed that all immunized mice developed neutralizing antibody responses, with titers ranging from 16 to 128 against CVA6-HeB (geometric mean titer [GMT] = 54) and from 32 to 256 against CVA6-TW141 (GMT = 76). In contrast, antibody titers in the PBS control group were below the limit of detection (Fig. 8B).

Second, we evaluated the protective efficacy of cHeB-E3238A against lethal CVA6 challenge. One-day-old ICR mice were primed with 0.5 ng of purified cHeB-E3238A virions or PBS, boosted on day 6 with 5 ng, and challenged on day 10 with the heterologous virulent strain CVA6-S0087b at an exceptionally stringent dose of 93,545 LD_50_ (where LD_50_ denotes the 50% lethal dose) (Fig. 8C). Note that CVA6-S0087b is non-cytopathic in cell culture but highly virulent in neonatal mice (8, 17). Following the challenge, all mice that received cHeB-E3238A survived the 14-day observation period without severe disease. In contrast, all mice in the PBS control group succumbed to the infection (Fig. 8D). This complete protection establishes the cHeB-E3238A mutant as an effective live-attenuated vaccine candidate against CVA6.

Histopathological analysis of limb muscles at 4 days post-challenge further revealed markedly attenuated pathology in vaccinated mice (Fig. 8E and F). The mean pathology score was significantly lower in the vaccinated group (Fig. 8E). PBS-control mice exhibited moderate to severe myositis, with three animals showing moderate and three showing severe muscle damage (Fig. 8F). Conversely, cHeB-E3238A-vaccinated mice displayed no to minimal pathology: seven of eight animals presented normal muscle architecture, and the remaining animal displayed only very mild myositis (Fig. 8F).

Collectively, these results establish that the cHeB-E3238A mutant serves as a highly effective live-attenuated vaccine candidate, conferring robust protection against a stringent heterologous challenge by completely preventing mortality and substantially mitigating tissue pathology.

### Genetic stability of the E3238A mutation during *in vivo* replication

A major safety concern for live-attenuated vaccines is the potential for reversion to virulence during replication in the host. This phenomenon is historically exemplified by the neurovirulent reversion of oral poliovirus vaccine (OPV) strains (18). To evaluate the genetic stability of the attenuating E3238A mutation, we assessed its fidelity following *in vivo* replication. Two-day-old neonatal mice were inoculated with the CVA6-cHeB-E3238A mutant virus. At 4 dpi, viral RNA was extracted from limb muscle tissues. The capsid region was then amplified by reverse transcription PCR (RT-PCR) and analyzed by Sanger sequencing.

In all tissue samples examined, sequencing confirmed that VP3 residue 238 remained alanine, with no evidence of reversion to the wild-type glutamic acid or emergence of other substitutions (Table 1). These results demonstrate that the E3238A mutation is genetically stable during *in vivo* replication, supporting the safety of this live-attenuated vaccine candidate. Nevertheless, longer-term studies in more tissues are needed to further confirm its stability.

**TABLE 1:** Genetic stability of the E3238A mutation during *in vivo* replication^*a*^

Mouse ID	VP3-238 codon	Amino acid	Reversion to WT (GAA, Glu)
#1	GCA	Ala	No
#2	GCA	Ala	No
#3	GCA	Ala	No
#4	GCA	Ala	No
#5	GCA	Ala	No

^*a*^ Two-day-old neonatal mice were inoculated with the CVA6-cHeB-E3238A mutant virus (5 ng/mouse). At 4 dpi, limb muscle tissues were collected for sequencing analysis.

## DISCUSSION

CVA6 has emerged as a major cause of severe HFMD, yet the specific viral determinants underlying its pathogenicity are not well understood. Here, through a systematic comparison of a lethal clinical isolate and an attenuated strain, we identify a single residue in the VP3 capsid protein as the critical switch governing lethal outcomes in a neonatal mouse model. This residue is dominant in circulating strains, and its mechanism involves modulating receptor engagement for efficient *in vivo* replication. Furthermore, targeted mutation of this site creates a stable, attenuated virus that serves as an effective live-attenuated vaccine candidate. Our work elucidates a key mechanism of CVA6 virulence and demonstrates a rational strategy for vaccine development.

### A single capsid residue acts as a master virulence switch

The initial observation that CVA6-TW141 and CVA6-HeB replicated with similar kinetics *in vitro* but exhibited profoundly different lethality *in vivo* (Fig. 1) pointed to genetic determinants affecting *in vivo* fitness. Our sequential mapping strategy—from chimeric viruses to a comprehensive panel of point mutants—definitively localized this determinant (Fig. 2 and 3). While the chimeric virus screen established the P1 capsid region as the primary locus for virulence (Fig. 2), finer mapping revealed that a single amino acid difference, VP3-E238, was responsible (Fig. 3). The evidence is definitive: introducing E238 into the attenuated CVA6-TW141 backbone (creating mutant A3238E) was sufficient to confer a 100% lethal phenotype (Fig. 3A through C). Conversely, reverting E238 to alanine in the otherwise lethal cHeB-P1 chimera (creating mutant E3238A) completely abolished mortality (Fig. 3D and E). This positions VP3-238 not merely as a contributor, but as a master molecular switch for CVA6 pathogenicity in this model.

Virulence determinants in other enteroviruses are often complex and involve multiple residues. For EV-A71, multiple capsid residues (VP1-98, VP1-145, and VP2-149) collectively influence virulence by modulating electrostatic surface charges and heparin-binding phenotype (19). In contrast, in CVA6, the VP3-E238 residue alone confers full virulence, while its mutation to alanine leads to complete attenuation, highlighting the potency of this single-residue switch.

### VP3-E238 represents a high-fitness, virulent phenotype in nature

The relevance of this laboratory finding to natural circulation is underscored by genomic epidemiology. Analysis of over 1,200 global CVA6 VP3 sequences revealed that glutamic acid (E) is the dominant residue at position 238, accounting for over 93% of strains (Fig. 4A). This strong conservation suggests a significant fitness advantage. Consistently, sequencing of the challenge strain used in our vaccine study, CVA6-S0087b, confirmed that it also carries the virulent VP3-E238 residue. Our functional testing of other natural variants (K, V, and Q) showed that none could match the full virulent potency of E238, especially at a lower challenge dose (Fig. 4C and D). This indicates that VP3-E238 is not an incidental polymorphism but a key component of a highly successful and virulent global lineage.

### Structural and functional context of VP3-238

The VP3 C-terminus, where residue 238 resides, represents a functionally important region in picornaviruses. Structural studies have demonstrated that this region contributes to the floor, wall, and rim of the canyon, a surface depression critical for receptor engagement (12). In the CVA10-KRM1 complex structure, the C-terminal residue T234 of VP3 directly contacts the KRM1 receptor (20). Similarly, in the CVA6-KRM1 complex structure, the VP3 C-terminal residue Q234 also participates in receptor interaction (12). Beyond receptor binding, the VP3 C-terminus is also involved in antibody recognition. For example, residues H240, A244, and Q247 in the VP3 C-terminus of EV-D68 form hydrogen bonds with the neutralizing MAb 2H12 (21). These findings collectively highlight the functional importance of the VP3 C-terminus. In CVA6, VP3-238 lies within this critical region. This provides a structural basis for its role in modulating KRM1 engagement and *in vivo* pathogenicity (Fig. 7).

### Mechanism of attenuation

A key finding was that the profound attenuation caused by the E3238A mutation (>10,000-fold change in lethal dose; Fig. 7A) occurred without detectable defects in basic virological properties *in vitro* (Fig. 5). The E3238A mutant assembled into morphologically normal particles with unaltered antigenicity and cell-binding capacity (Fig. 5). The attenuation mechanism was instead linked to a specific step critical *in vivo*. We found that the E3238A mutant had a markedly reduced binding affinity for the murine entry receptor KRM1 (Fig. 7C). This impaired receptor interaction provides a direct mechanistic explanation for the observed *in vivo* phenotype: significantly lower viral loads in key target tissues like limb muscle and the spinal cord (Fig. 6B and 7B), leading to attenuated pathology (Fig. 6C and D) and the absence of severe clinical disease (Fig. 6A and 7A).

### From pathogenic determinant to vaccine candidate

Our vaccine design intentionally utilized the CVA6-HeB capsid backbone, as opposed to strains like CVA6-TW141, for a key structural reason. Unlike TW141, which primarily forms expanded “A-particles” with suboptimal antigenicity, purified CVA6-HeB consists almost exclusively of mature, compact virions (12). Compared to expanded A-particles, the compact conformation of the mature viral capsid presents a broader array of conformational neutralizing epitopes and mediates superior binding to the entry receptor KREMEN1 (12). A vaccine platform based on this mature virion structure is therefore optimal for eliciting a potent, protective antibody response. Other naturally occurring VP3-238A isolates would require experimental validation of their virulence, capsid conformation, immunogenicity, and protective efficacy before they could be considered as vaccine candidates.

The ideal live-attenuated vaccine candidate is highly attenuated yet immunogenic. The cHeB-E3238A mutant perfectly embodies this principle. Its complete avirulence (Fig. 6 and 7) ensures safety, while its preserved antigenic structure (Fig. 5C through E) enables the induction of protective immunity. Our proof-of-concept vaccination study yielded highly robust results. A two-dose regimen of cHeB-E3238A conferred complete protection against a subsequent lethal challenge with a heterologous, highly virulent CVA6 strain (Fig. 8). The protective effect extended beyond survival and disease signs to histopathology, with vaccinated animals showing absent-to-minimal muscle damage post-challenge versus severe pathology in controls (Fig. 8).

While the neonatal immune system is developmentally distinct from that of adults, studies have shown that T cells and B cells appear in the mouse spleen as early as embryonic day 15 and 16, and antigen-binding cells are present at birth, with the capacity for antibody synthesis arising at approximately 2 weeks of age (22). Consistent with this time frame, our two-dose immunization regimen (days 1 and 6) elicited neutralizing antibody responses in all immunized mice by day 13 (Fig. 8B), confirming that the cHeB-E3238A vaccine effectively activates the neonatal immune system and confers protection.

Sequence analysis of the heterologous challenge strain CVA6-S0087b revealed that its P1 capsid region shares 97.93% amino acid identity with the vaccine backbone CVA6-HeB, with 17 divergent residues. This indicates a certain degree of sequence divergence. Notably, despite these capsid differences, the cHeB-E3238A vaccine still provided robust protection against lethal challenge with CVA6-S0087b (Fig. 8), demonstrating its potential to protect against genetically diverse circulating CVA6 strains.

This demonstrates that rational attenuation via the VP3-E238 site disrupts pathogenicity without compromising immunogenicity, validating this genetic modification as a powerful strategy for vaccine design.

### Conclusion

In summary, this study identifies VP3-E238 as a central genetic determinant of CVA6 virulence. Its mechanism involves optimizing capsid-receptor (KRM1) engagement, which is crucial for efficient *in vivo* replication and pathogenesis. From an applied perspective, the E3238A mutation provides a precise and stable method for generating attenuated CVA6 strains. The efficacy of the cHeB-E3238A mutant as a protective live-attenuated vaccine candidate establishes a direct translational pathway from mechanistic discovery to public health intervention. This work not only advances the fundamental understanding of CVA6 biology but also delivers a promising candidate for preventing severe disease caused by this increasingly prevalent virus.

## MATERIALS AND METHODS

### Cells and viruses

Human rhabdomyosarcoma (RD) and human embryonic kidney (HEK) 293T cells were cultured in Dulbecco’s Modified Eagle Medium (DMEM, Gibco) supplemented with 10% fetal bovine serum (FBS) at 37°C.

The CVA6-HeB strain (clinical isolate 54203/HeB/CHN/2012; GenBank MK106189) (16) was obtained from Dr. Yong Zhang’s laboratory and propagated in RD cells. An early-passage CVA6-HeB virus stock was used for all experiments, and its consensus sequence was confirmed to be identical to the GenBank entry. The CVA6-TW141 strain (TW-2007-00141; GenBank KR706309) was rescued from an infectious clone (provided by Professor Tong Cheng, Xiamen University) (15) and likewise propagated in RD cells. Viral titers were determined by 50% tissue culture infectious dose (TCID_50_) assay on RD cells.

### Proteins and antibodies

The recombinant mouse KRM1 ectodomain (residues A23–G373) fused to a human IgG1 Fc tag (mKrm1-Fc) was expressed in HEK 293F cells and purified as previously described (23).

Polyclonal rabbit antiserum against CVA6 VP1 was generated by immunization with *Escherichia coli*-expressed recombinant VP1 protein emulsified in Freund’s adjuvants (8). Polyclonal mouse anti-CVA6 serum was obtained by immunizing BALB/c mice with alum-adjuvanted, purified CVA6-HeB virions (12). The CVA6-specific neutralizing MAb 3H7 was generated and characterized in our earlier study (12).

### Viral growth kinetics

RD or HEK293T cells seeded in 24-well plates were infected with CVA6-HeB or CVA6-TW141 at an MOI of 0.1 for 1 h at 37°C. After washing twice with PBS, cells were maintained in fresh DMEM containing 1% FBS. At the indicated time points (0, 24, and 48 h post-infection), both cells and culture supernatants were harvested, subjected to two freeze–thaw cycles, and clarified by centrifugation. Viral titers in the supernatants were determined by TCID_50_ assay on RD cells.

### Western blot analysis

Protein samples were denatured in SDS loading buffer at 100°C for 10–30 min, separated by SDS-PAGE, and transferred onto PVDF membranes. The membranes were incubated with horseradish peroxidase (HRP)-conjugated anti-β-actin antibody (Proteintech) or rabbit anti-CVA6 VP1 polyclonal antibody. For the latter, membranes were subsequently probed with an HRP-conjugated goat anti-rabbit secondary antibody (Proteintech). Protein bands were detected using enhanced chemiluminescence (ECL) substrate and visualized by chemiluminescence imaging.

### Mouse infection model for virulence assessment

To assess virulence *in vivo*, 2-day-old ICR mice were inoculated i.p. with the indicated virus at the specified dose (as detailed in the respective figure legends). Mice were monitored daily for 14 days for survival and clinical signs of disease. Clinical scores were assigned as follows: 0, healthy; 1, reduced mobility; 2, limb weakness; 3, limb paralysis; and 4, death.

### Construction and rescue of chimeric CVA6 viruses

To map virulence determinants, chimeric viruses were constructed on the CVA6-TW141 infectious clone backbone. First, total viral RNA was extracted from the CVA6-HeB strain using Trizol reagent (Vazyme, China). The RNA was then reverse-transcribed into cDNA using the HiScript III 1st Strand cDNA Synthesis Kit (Vazyme, China). Subsequently, individual genomic regions (5′ UTR, P1 capsid, P2, P3, or 3′ UTR) were amplified from the cDNA template by PCR. Each amplified fragment was assembled into the corresponding site of the linearized CVA6-TW141 backbone plasmid using 2X MultiF Seamless Assembly Mix (ABclonal, China), generating a panel of chimeric infectious clones (designated cHeB-5′UTR, cHeB-P1, cHeB-P2, cHeB-P3, and cHeB-3′UTR).

For virus rescue, HEK 293T cells seeded in 24-well plates were co-transfected with a chimeric plasmid and a T7 RNA polymerase expression plasmid using Lipofectamine 2000 (Gibco). At 72 h post-transfection, the culture supernatant was harvested. The rescued virus was amplified in RD cells. The genomic integrity was confirmed by Sanger sequencing after reverse transcription PCR (RT-PCR) amplification. Final virus stocks were titrated on RD cells using the TCID_50_ assay and subsequently assessed for virulence in neonatal mice as described above.

### Construction and rescue of CVA6 mutant viruses

To identify specific virulence determinants, site-directed mutagenesis was performed on the CVA6-TW141 infectious clone backbone using 2X MultiF Seamless Assembly Mix (ABclonal). A panel of 15 mutant clones (Mutants #1 to #15) was generated to systematically test the 19 amino acid differences in the P1 region between CVA6-HeB and CVA6-TW141, as summarized in Fig. 3A. Separately, the attenuating VP3-E238A (E3238A) mutation was introduced into the virulent chimeric clone cHeB-P1 (designated cHeB-WT) using the same assembly strategy, generating the mutant cHeB-E3238A. Subsequently, to assess the functional impact of natural variations at VP3-238, four additional point mutants (A3238E, A3238V, A3238K, and A3238Q) were constructed on the CVA6-TW141 backbone, reproducing the amino acids found in circulating strains.

All constructed mutations were confirmed by Sanger sequencing of the targeted regions. The mutant viruses were then rescued, amplified, titrated, and assessed for virulence in neonatal mice following the standard protocols described above.

### Sequence conservation analysis

To evaluate natural variation at VP3 residue 238, we retrieved all available VP3 protein sequences of CVA6 from the NCBI non-redundant protein database using BLASTP (search date: August 2025). A total of 1,276 sequences were obtained. Multiple sequence alignment was performed using CLC Sequence Viewer (v8.0). The amino acid identity and frequency at VP3 position 238 were analyzed.

### Purification of CVA6 cHeB-WT and cHeB-E3238A virions

To obtain highly purified virions for downstream analyses, the rescued cHeB-WT and cHeB-E3238A viruses were amplified in large-scale RD cell cultures (500 mL each) and cultured for 3 days. The infected cell cultures were subjected to two freeze–thaw cycles and clarified by centrifugation. Viruses in the clarified lysate were precipitated overnight at 4°C with 10% polyethylene glycol (PEG) 8000 and 200 mM NaCl. The precipitate was collected by centrifugation, resuspended in 0.15 M PBS, and clarified again by high-speed centrifugation. The concentrated virus solution was then layered onto a 20% sucrose cushion and ultracentrifuged at 27,000 rpm for 4 h. The pellet was resuspended in PBS and further purified by ultracentrifugation on a 10%–50% (wt/vol) sucrose gradient at 39,000 rpm for 3 h. Twelve fractions were collected from the gradient and analyzed by SDS-PAGE to identify those containing mature virions, which were used for subsequent characterization.

### Negative-stain electron microscopy

Purified CVA6 cHeB-WT and cHeB-E3238A virions (from fraction #10) were diluted to 50 μg/mL in PBS, applied to glow-discharged carbon-coated copper grids, and stained with 2% uranyl acetate. Images were acquired using a Tecnai G2 Spirit transmission electron microscopy (FEI, USA) operated at an accelerating voltage of 200 kV.

### Antigenicity analysis by ELISA

Purified cHeB-WT and cHeB-E3238A virions were serially diluted in PBS, coated onto 96-well plates overnight at 4°C, and then blocked with 5% (wt/vol) skim milk in PBS containing 0.05% Tween-20 (PBST). The plates were incubated for 2 h at 37°C with one of the following primary antibodies: a 1:5,000 dilution of mouse anti-CVA6 polyclonal serum or 50 ng/well of the mouse neutralizing MAb 3H7 (12). After washing with PBST, bound antibodies were detected by incubation for 1 h at 37°C with an HRP-conjugated goat anti-mouse IgG secondary antibody (Proteintech). Following final washes, the reaction was developed with TMB substrate. The reaction was stopped, and the absorbance was measured at 450 nm using a microplate reader.

### Virus attachment assay

Pre-chilled monolayers of RD cells in 24-well plates were incubated with 500 µL/well of purified CVA6 virions (1  ×  10⁹ copies/mL) at 4°C for 2 h to allow binding. Following incubation, the unbound virus was removed by washing twice with ice-cold PBS. Total RNA was immediately extracted from the cells using Trizol reagent (Vazyme, China) and reverse transcribed into cDNA using the PrimeScript RT reagent Kit (Takara). The amount of cell-associated viral RNA was quantified by quantitative RT-PCR (RT-qPCR) using SYBR Premix Ex Taq (Takara) on a LightCycler 480 II instrument (Roche). Viral RNA levels were normalized to the endogenous reference gene β-actin.

The primer sequences used were as follows: CVA6: Forward, 5′-TACTTTGGGTGTCCGTGTTT-3′; Reverse, 5′-TGGCCAATCCAATAGCTATATG-3′ (10). β-actin: Forward, 5′-GGACTTCGAGCAAGAGATGG-3′; Reverse, 5′-AGCACTGTGTTGGCGTACAG-3′. Relative quantification was performed using the 2^−ΔΔCt^ method.

### Quantification of tissue viral loads

To determine viral replication *in vivo*, infected mice were euthanized at 4 dpi. Limb muscle, spinal cord, and brain were aseptically collected, weighed, and homogenized in 400 μL/sample of DMEM supplemented with 1% FBS using a tissue homogenizer. Tissue homogenates were clarified by high-speed centrifugation at 4°C. Viral titers in the clarified tissue supernatants were quantified by TCID_50_ assay on RD cells. Titers were calculated and expressed as TCID_50_ per gram of tissue (TCID_50_/g).

### Histopathological analysis

Hindlimb skeletal muscle tissues were collected from mice at 4 days post-infection or post-challenge. Tissues were fixed in 4% paraformaldehyde, processed through standard dehydration, embedded in paraffin, and sectioned. Sections were stained with hematoxylin and eosin (H&E) by Servicebio Technology Company (China). Stained sections were examined under a light microscope. Histopathological changes were scored on a semi-quantitative scale: 0, no pathology; 1, mild; 2, moderate; and 3, severe damage.

### Analysis of murine KRM1 receptor binding by ELISA

Purified CVA6 cHeB-WT and cHeB-E3238A virions were serially diluted in PBS, coated onto 96-well plates overnight at 4°C, and then blocked with 5% skim milk in PBS. The plates were incubated with 100 ng/well of recombinant mKrm1-Fc protein (23) for 1 h at room temperature. After washing with PBST, bound mKrm1-Fc was detected by incubation with an HRP-conjugated goat anti-human IgG antibody (Proteintech) for 1 h at room temperature. Following a final wash, the reaction was developed with TMB substrate. The reaction was stopped, and absorbance was measured at 450 nm.

### Structural localization

The structural model of the CVA6-HeB mature virion (PDB: 9VFQ) (12) was used to map the location of VP3 residue 238. Visualization, analysis, and rendering of the capsid protomer were performed using UCSF Chimera (version 1.15).

### Immunization and neutralization assay

To evaluate the immunogenicity of the cHeB-E3238A vaccine candidate, 1-day-old ICR mice were immunized i.p. with purified cHeB-E3238A virions (0.5 ng/pup on day 1 and 5 ng/pup on day 6) or PBS as a control. Blood samples were collected on day 13, and sera were heat-inactivated at 56°C for 30 min.

Neutralizing antibody titers were determined by a standard microneutralization assay. Briefly, serial twofold dilutions of serum (starting at 1:16) were mixed with 100 TCID_50_ of CVA6-HeB or CVA6-TW141 and incubated at 37°C for 1 h. The mixtures were then added to RD cell monolayers in 96-well plates and incubated at 37°C for 3–4 days. The neutralizing titer was defined as the highest serum dilution that completely inhibited virus-induced cytopathic effect (CPE).

### Vaccination and challenge study

To evaluate vaccine efficacy, 1-day-old ICR mice were randomly assigned to receive either the vaccine candidate (cHeB-E3238A) or PBS (control). The vaccinated group was immunized i.p. with 0.5 ng of purified cHeB-E3238A virions, followed by a booster of 5 ng on day 6; the control group received PBS at the same time points.

On day 10, all mice were challenged i.p. with a lethal dose of the heterologous virulent strain CVA6-S0087b. This challenge strain lacks detectable CPE *in vitro*. Therefore, to standardize the inoculum, its 50% lethal dose (LD_50_) was first determined in 10-day-old ICR mice. The administered challenge dose was 93,545 LD_50_. Following the challenge, survival and clinical signs were monitored daily for 14 days using the established scoring criteria.

For histopathological assessment, a separate cohort of mice was euthanized at 4 days post-challenge. Limb muscle tissues were collected and processed for H&E staining and analysis as described above.

### Statistical analysis

All statistical analyses were performed using GraphPad Prism version 8.

## Data Availability

All relevant data are provided as figures within the paper. More detailed methods are available from the corresponding author upon request.
